# Supplementary material for: Magnetically-dressed CrSBr exciton-polaritons in ultrastrong coupling regime
Source: Nat Commun. 2023 Sep 25;14:5966. doi: 10.1038/s41467-023-41688-7 (PMC10520032; doi:10.1038/s41467-023-41688-7)
Supplement: Supplementary file 1 — Supplementary Information [file 41467_2023_41688_MOESM1_ESM.pdf]

Supplementary Information for
Magnetically-dressed CrSBr exciton-polaritons in ultrastrong coupling regime

Tingting Wang[1,2,⋆], Dingyang Zhang[1,⋆], Shiqi Yang[1,3,⋆], Zhongchong Lin[1], Quan Chen[4], Jinbo Yang[1], Qihuang Gong[1,5,6], Zuxin Chen[4,†], Yu Ye[1,2,5,6,†] and Wenjing Liu[1,5,7,†]

[1]State Key Laboratory for Mesoscopic Physics and Frontiers Science Center for Nano-optoelectronics, School of Physics, Peking University, Beijing 100871, China

[2]Collaborative Innovation Center of Quantum Matter, Beijing 100871, China

[3]Academy for Advanced Interdisciplinary Studies, Peking University, Beijing 100871, China

[4]School of Semiconductor Science and Technology, South China Normal University, Foshan, 528225, China

[5]Yangtze Delta Institute of Optoelectronics, Peking University, Nantong 226010, China

[6]Liaoning Academy of Materials, Shenyang, 110167, China

[7]Collaborative Innovation Center of Extreme Optics, Shanxi University, Taiyuan, 030006, China

[⋆]These authors contributed equally

[†]Corresponding author: chenzuxin@m.scnu.edu.cn, ye_yu@pku.edu.cn, wenjingl@pku.edu.cn

1. Data analysis

The transfer matrix method (TMM) was applied to calculate the reflectance of the multilayer thin film structures of the cavity-coupled CrSBr. Fig. S1 presents the experimentally measured reflectance spectra (blue curves) of three CrSBr flakes with different thicknesses and their corresponding TMM fittings (red curves).

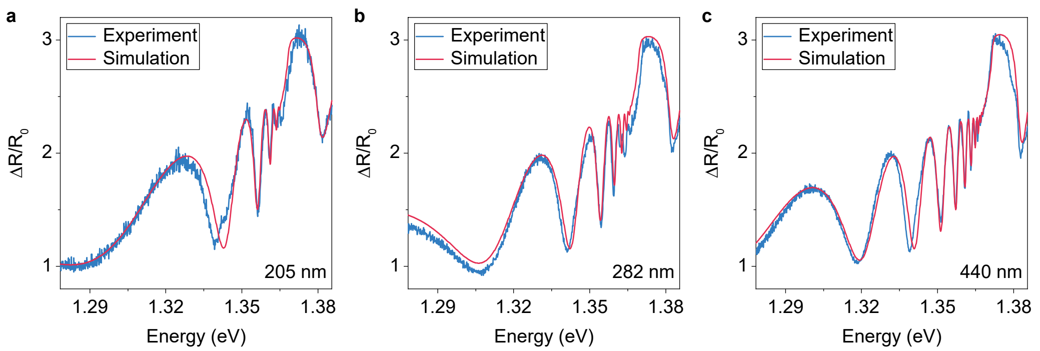

Fig. S1: **Reflectance spectra of CrSBr flakes on Si substrates. a-c.** Reflectance spectra of CrSBr flakes with thicknesses of 205 nm(**a**), 282 nm(**b**), and 440 nm(**c**) with corresponding TMM fittings. The parameters applied in the fitting are **a.** $\varepsilon_{\mathrm{bg}} = 11.0$, $\omega_{\mathrm{X}} = 1.367$ eV, $\Gamma_{\mathrm{X}} = 0.68$ meV, $f_{\mathrm{X}} = 2.00$ (eV)^2, $\omega_{\mathrm{X}^*} = 1.385$ eV, $\Gamma_{\mathrm{X}^*} = 7.5$ meV, $f_{\mathrm{X}^*} = 0.65$ (eV)^2; **b.** $\varepsilon_{\mathrm{bg}} = 11.0$, $\omega_{\mathrm{X}} = 1.370$ eV, $\Gamma_{\mathrm{X}} = 1.0$ meV, $f_{\mathrm{X}} = 2.03$ (eV)^2, $\omega_{\mathrm{X}^*} = 1.386$ eV, $\Gamma_{\mathrm{X}^*} = 6.0$ meV, $f_{\mathrm{X}^*} = 0.53$ (eV)^2; **c.** $\varepsilon_{\mathrm{bg}} = 11.0$, $\omega_{\mathrm{X}} = 1.372$ eV, $\Gamma_{\mathrm{X}} = 0.70$ meV, $f_{\mathrm{X}} = 2.00$ (eV)^2, $\omega_{\mathrm{X}^*} = 1.386$ eV, $\Gamma_{\mathrm{X}^*} = 5.0$ meV, $f_{\mathrm{X}^*} = 0.60$ (eV)^2.

Fig. S2 presents the finite-difference time-domain (FDTD) simulation result compared with the experimental reflectance spectrum as shown in Fig. 2b in the main text. In general, the simulation nicely reproduces the experiments. Its deviation from the experimental data at large angles is due to the large anisotropy of CrSBr and the tilting between its crystalline axis and the incident plane.

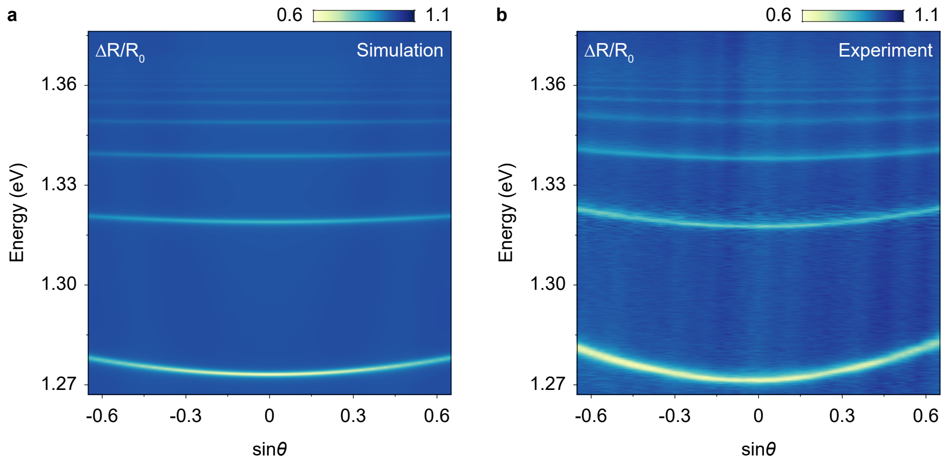

Fig. S2: **FDTD simulation of the angle-resolved reflectance spectrum of the cavity-coupled CrSBr. a.** The FDTD simulation of the angle-resolved reflectance spectrum as shown in Fig. 2b. The simulated structure was excited by an angle-varying plane-wave source, where a far-field power monitor was implemented to measure the reflected signal. **b.** The experimentally measured angle-resolved reflectance spectrum same as in Fig. 2b. The deviation between the simulation and the experimental data at large angles is due to the large anisotropy of CrSBr and the tilting between its crystalline axis and the incident plane.

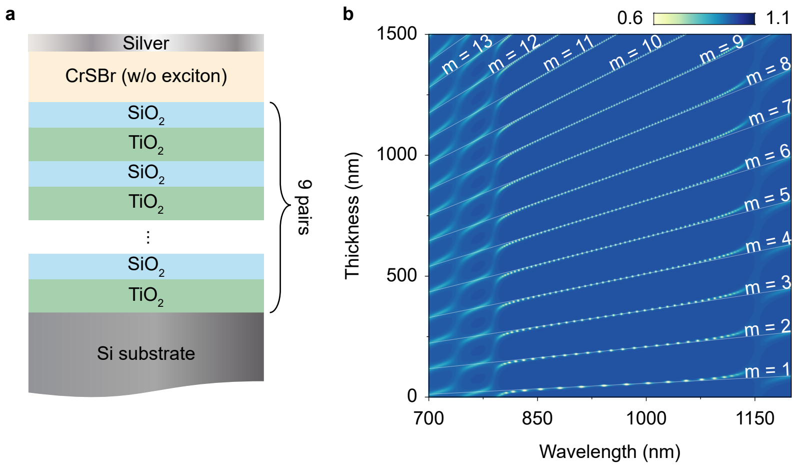

Fig. S3: **Dispersion relation of the bare photonic modes of the microcavity. a.** The illustration of the layer structure of the Tamm plasmon microcavity. The cavity is composed of (from top to bottom) 45 nm thick silver, a thickness-varying dielectric layer (CrSBr without excitons) with $\varepsilon = \varepsilon_{\mathrm{bg}} = 11$, and 9 pairs of 160 nm SiO_2/100 nm TiO_2 on Si substrate. **b.** The Tamm plasmon modes were calculated through the TMM while sweeping the thickness of the dielectric CrSBr layer in a 5 nm interval. White lines: linear fit of the cavity mode dispersion for different mode numbers of m.

Fig. S3 shows the bare photonic cavity modes by calculating the Tamm plasmon mode dispersions while replacing the CrSBr flake with a dielectric layer with $\varepsilon = \varepsilon_{\mathrm{bg}} = 11$. According to the electric field distribution, the modes were assigned to different mode families with the mode numbers m labeled in Fig. S3b. Each mode family was fitted to a linear dispersion relation, from which the photonic mode energies associated with the CrSBr thickness were calculated.

2. Magnetic characterizations of bulk CrSBr

The magnetic properties of the bulk CrSBr were studied via the vibrating sample magnetometer measurements. The temperature-dependent magnetic susceptibility of zero-field cooling with a field of 0.1 T applied along different axes is shown in Fig. S4a, revealing the value of T_N is about 132 K. Besides, the magnetic moment (M) versus the applied magnetic field ($\mu_0 H$) along different axes at three selected temperature are shown in Fig. S4b-d. At a temperature of 150 K above T_N, the M-$\mu_0 H$ curve shows the identical paramagnetic response in all three crystallographic axis directions. At temperatures below T_N, CrSBr undergoes spin canting in the hard axis directions (a and c axes) and spin flipping in the easy axis direction (b axis), finally reaching the forced-FM states.

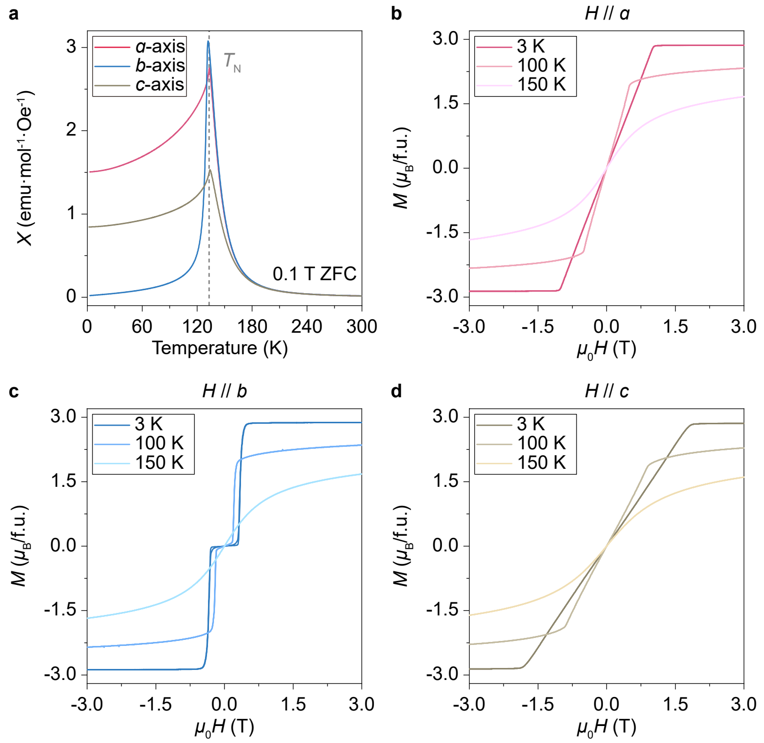

Fig. S4: **Magnetic characterizations of CrSBr bulk crystal. a.** Magnetic susceptibility versus temperature along the a axis (blush line), b axis (steel blue line), and c axis (dark tan line) under zero-field cooling at 0.1 T, where the Néel temperature (T_N) can be clearly identified. **b-d.** The magnetic moment (M) of the CrSBr bulk crystal versus the applied magnetic field ($\mu_0 H$) oriented along the a axis (**b.**), b axis (**c.**) and c axis (**d.**) at three selected temperature of 3 K, 100 K, and 150 K. At a temperature of 150 K above T_N, the M-$\mu_0 H$ curve show identical paramagnetic response in all three crystallographic axis directions. At temperatures below T_N, CrSBr undergoes spin canting in the hard axis directions (a and c axes) and spin flipping in the easy axis direction (b axis), finally reaching the forced-FM states.

3. Morphological characterizations of the CrSBr flake in Fig. 3 in the main text

The lateral dimensions of the CrSBr flake were measured via optical microscope, showing sizes over 60 μm in both length and width. The flake thickness was characterized by the atomic force microscope and extracted to be 460 nm, as presented in Fig. **S5**.

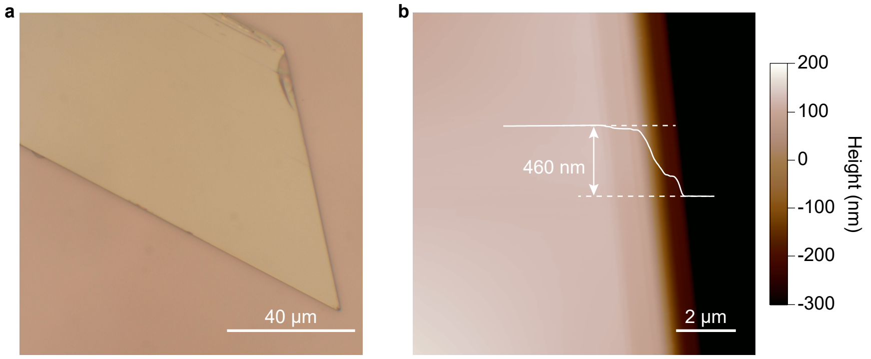

Fig. S5: **Morphological characterizations of the CrSBr flake in Fig. 3.** **a.** The optical microscopic image. **b.** The sample thickness was measured by the atomic force microscope, showing a thickness of 460 nm.

4. Comparison of exciton-photon coupling between bare CrSBr flake and Tamm plasmon cavity sample

The same reflectance measurements were conducted on a bare CrSBr flake (thickness of 620 nm) placed on SiO_2/Si substrate without an external microcavity (Fig. S6). Interestingly, the multiple branches shown in the reflectance spectrum in Fig. S6a indicate that the thick CrSBr flake itself can serve as an intrinsic microcavity that supports dielectric leaky modes, which in turn leads to the emergence of exciton-polaritons. Meanwhile, the evolution behavior of the temperature-dependent reflectance spectrum of the bare CrSBr flakes is consistent with that of the Tamm plasmon microcavity sample shown in Fig. 3 in the main text. Similarly, TMM simulation is utilized to calculate the background permittivity $\varepsilon_{\mathrm{bg}}$, exciton energy ω_{ex}, and the square root of the exciton oscillator strength $\sqrt{f_{\mathrm{ex}}}$ as the functions of temperature, where consistent results are obtained (Fig. S6b). Furthermore, to highlight the role of the Tamm plasmon cavity, we applied FDTD simulations to compare the performance in them of coupling strength, mode quality factor, field distribution, etc., with and without the cavity.

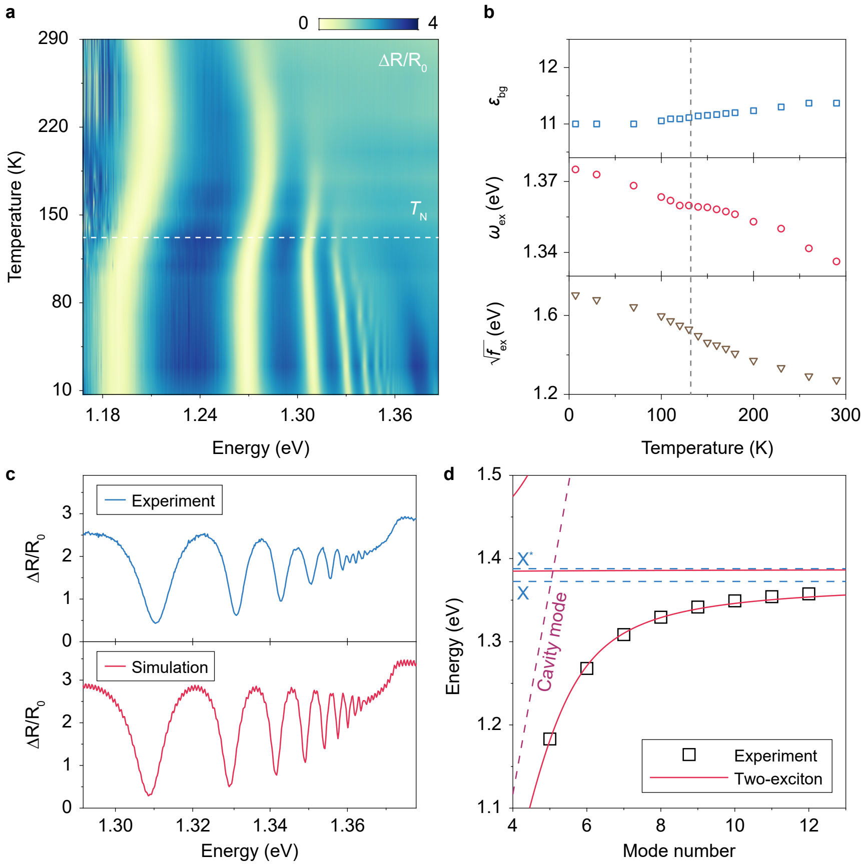

Fig. S6: **Optical characterizations of CrSBr flake without an external microcavity. a.** Temperature-dependent reflectance spectrum of a CrSBr flake (thickness of 620 nm) on a 285 nm SiO_2/Si substrate at normal incidence. The reference reflection R_0 is measured on the same SiO_2/Si substrate. **b.** The TMM calculated background permittivity $\varepsilon_{\mathrm{bg}}$, exciton energy ω_{ex}, and the square root of the exciton oscillator strength $\sqrt{f_{\mathrm{ex}}}$ as the functions of temperature. In the calculation, the one-exciton Lorentzian model is applied. **c.** Reflectance spectrum polarized along the b axis at normal incidence and its corresponding FDTD simulation results. **d.** Mode number-dependent exciton-polariton energy and polariton dispersion fitted using the COM two-exciton Lorentzian model.

The coupling strengths of the bare CrSBr flake are shown in Fig. S6c-d. The fitting procedure is the same as that

in Fig. 2 in the main text, where a two-exciton model is used for FDTD simulations ($\varepsilon_{\text{bg}} = 11.0$, $f_\text{X} = 2.37$ (eV)^2, $f_{\text{X}^*} = 0.5$ (eV)^2, $\Gamma_\text{X} = 1.0$ meV, and $\Gamma_{\text{X}^*} = 5.0$ meV) and compared with the experimental reflectance spectrum, as shown in Fig. S6c to confirm a good match. The mode number of each mode was then identified by the calculated spatial field distribution. The corresponding photonic mode dispersion was simulated by replacing CrSBr with a dielectric with $\varepsilon_{\text{bg}} = 11.0$. The fitted coupling strength is $g_X = 170$ meV, which is almost the same as 169 meV of the sample in Fig. 2 in the main text, despite the fact that the CrSBr flake here is much thicker (620 nm) and of larger exciton oscillator strength ($f_\text{X} = 2.37$ (eV)^2) comparing to the one used in Fig. 2 (460 nm thick, $f_\text{X} = 2.00$ (eV)^2). Besides, we also calculated the coupling strength of a hypothetical CrSBr flake on the SiO_2/Si substrate, with the same thickness and material parameters as those in Fig. 2, and obtained a smaller coupling strength of $g_X = 154$ meV.

A properly designed external cavity can significantly enhance the performance of exciton-polaritons by improving both the temporal and spatial confinements. FDTD simulations were applied to compare the quality factor and field distribution of the photonic modes of the Tamm plasmon cavity and the SiO_2/Si substrate, as shown in Fig. S7. For both cases, the thickness and refractive index of the CrSBr flake (without exciton) are taken to be 460 nm and $\varepsilon_{\text{bg}} = 11$, respectively, as in the main text. The simulated reflectance spectra of the two configurations are shown in Fig. S7, with quality factors of 410 and 12 extracted for the Tamm cavity and no cavity cases. Furthermore, the spatial distribution of the electric field of their photonic modes is calculated and plotted, as shown in Fig. S7b. With a Tamm cavity, the electric field is mostly confined in the CrSBr flake, whereas without a cavity, the mode leaks significantly into the SiO_2 layer and into the air. These differences suggest that the external cavity can enhance the coupling strength and lifetime of the polaritons through better spatial and temporal confinements, which may be advantageous in polariton lasing, condensation, and other optoelectronic applications.

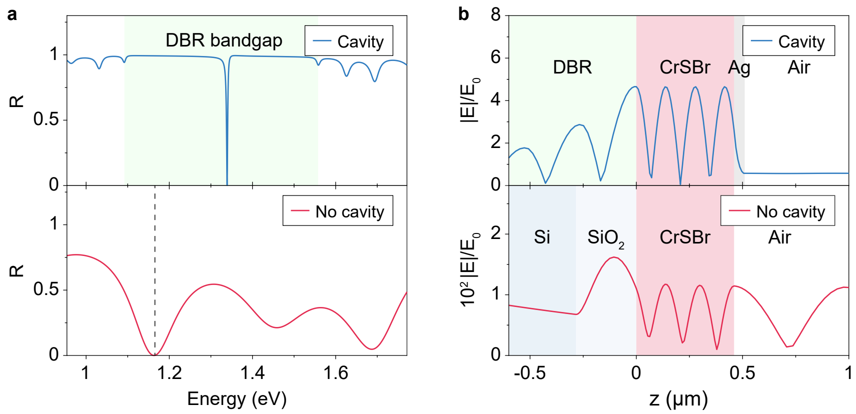

Fig. S7: **Comparison of photonic modes with and without the Tamm plasmon cavity. a.** Simulated reflectance spectra of a 460 nm dielectric layer sandwiched in a Tamm plasmon cavity (top) and placed on a SiO_2/Si substrate (bottom). Photonic modes are identified as dips in the reflectance spectra. **b.** Electric field amplitude distributions for the Tamm plasmon mode (top) and dielectric leaky mode (bottom), where the dielectric leaky mode is marked by the vertical dashed line in the bottom panel of **a**. In the calculations, the permittivity of the dielectric layer is taken as the background permittivity of CrSBr $\varepsilon_{\text{bg}} = 11.0$.
