# Supplementary material for: Magnetically-dressed CrSBr exciton-polaritons in ultrastrong coupling regime
Source: Nat Commun. 2023 Sep 25;14:5966. doi: 10.1038/s41467-023-41688-7 (PMC10520032; doi:10.1038/s41467-023-41688-7)
Supplement: Supplementary file 3 — Source Data [file 41467_2023_41688_MOESM3_ESM.zip › Source data guide.docx]

The Source Data ZIP file includes four source data files (**Figure 1.xlsx, Figure 2.xlsx, Figure 3.xlsx and Figure 4.xlsx**).

- Legend for **Figure 1.xlsx** file**:**

This file includes source data for Figure 1 in the main text.

- Legend for **Figure 2.xlsx** file**:**

This file includes source data for Figure 2 in the main text.

- Legend for **Figure 3.xlsx** file**:**

This file includes source data for Figure 3 in the main text.

- Legend for **Figure 4.xlsx** file**:**

This file includes source data for Figure 4 in the main text.

Different sheets correspond to different panels.
